# AI for Not Bad

**DOI:** 10.3389/fdata.2019.00032

**Published:** 2019-09-11

**Authors:** Jared Moore

**Affiliations:** Wadhwani Institute for Artificial Intelligence, Mumbai, India

**Keywords:** artificial intelligence, social good, not bad, ethics, data science, critical reflection

## Abstract

Hype surrounds the promotions, aspirations, and notions of “artificial intelligence (AI) for social good” and its related permutations. These terms, as used in data science and particularly in public discourse, are vague. Far from being irrelevant to data scientists or practitioners of AI, the terms create the public notion of the systems built. Through a critical reflection, I explore how notions of AI for social good are vague, offer insufficient criteria for judgement, and elide the externalities and structural interdependence of AI systems. Instead, the field known as “AI for social good” is best understood and referred to as “AI for not bad.”

## Introduction

We have begun to apply artificial intelligence (AI) to areas that claim to interact with “social good.” New academic centers and initiatives label themselves as such. Cornell and Berkeley work on human-compatible AI^1^ and Stanford's Human-Centered AI initiative aims “to advance AI research, education, policy, and practice to improve the human condition.”^2^ The University of Hong Kong claims to work on “beneficial AI.”^3^ The University of Washington and the University of Chicago offer programs on “data science for social good,”^4^ while Harvard and the University of Southern California call it “AI for social good.”^5^

These efforts carry over into conferences. At the prestigious AI conferences NeurIPS, ICML, and ICLR this past year, one group led workshops on “AI for social good.”^6^ Bloomberg News has held an annual “Data for good exchange” conference since sponsoring a “special event” at ACM KDD in 2014, a year where the overall conference had the theme “Data Science for Social Good,” defined as “applying data science to improve civic and social outcomes.”^7^ A 2018 talk at ACM SIGIR used same term (Ghani, 2018) and is similar to non-academic conferences like “AI on a social mission”^8^ and the “Rework AI for Good Summit.”^9^ Philosophers, too, have asked, “For The Public Good? Values and Accountability in AI and Data Science.”^10^

The world outside of universities has not been quiet. Google, Facebook, IBM, and Intel have pages on “AI for social good”^11^ and Microsoft has one about “AI for good.”^12^ AI research labs like AI2, WadhwaniAI, and MILA, respectively discuss AI for “common good,” “social good,” and “humanity.”^13^ Government initiatives from India, the U.S., and China do similarly^14^.

“Social good” shifts between social responsibility, societal impacts, society, common good, the good, development, and ethics. Its proposals come in similar forms: calls for more data, better data, broader application, more diverse voices, reflexivity, transparency, changes to funding priorities, more education, more regulation—more.

The meaning of artificial intelligence shifts as well. It may mean “algorithmic systems,” or “automated decision making” (Harris and Davenport, 2005)—other times, it is synonymous with “data science” or “big data.” It also could be the case that AI does not truly exist and only refers to some yet-to-come future (Walch, 2018) when, presumably, this “social good” will actually be achieved. To others, that AI does not exist is misleading (Schank, 1987; Bringsjord and Schimanski, 2003). To such technical minds, AI would chiefly refer to a set of techniques like machine learning, deep learning, active learning, or reinforcement learning^15^.

“AI for the good” de-politicizes the problems addressed. Many of these problems, like poverty, recidivism, and the distribution of resources, are ones of institutional failure. Technology-based approaches, when not aimed at the root of problems, divert attention from the proper recourse: structural change.

In this paper, I offer a critical perspective on the use of language of AI practitioners like myself who, from practice to theory, apply their work to some definition of “good.” I use discursive analysis to explore the space between the notion of such projects and their actuality. In so doing, I follow Green (2018) in identifying AI systems as inherently political. Vague terms are the wagons of a modern gold rush into the promised riches of a mythic AI frontier. Like the California gold rush, this expansion may bring environmental degradation, concentrations rather than distributions of wealth, and the oppression of marginalized populations.

It is not the primary aim of this paper to synthesize a definition of AI, social good, or their combination. Chiefly, I theorize about what the apparent use omits. Nonetheless, I do offer and argue for a preliminary definition of good in section three. I use the term “data science” to loosely denote AI systems. For clarity hereafter and unless otherwise noted, “AI for the good” or “AI for social good” will encompass the above uses as they exist today and will refer to the projection of the computational discipline onto some definition of public or societal good. AI itself means, and will be used to mean in this paper, more than just the application of a statistical model like logistic regression to a dataset: it will mean the notions associated with such systems, the specifics of which I will explore below.

This paper proceeds in four parts. First, I review relevant literature. Second, I argue why “AI for the good,” as it is used, is inappropriate. Third, I address possible critiques of my approach. Fourth, I suggest directions for those who aim to work in “AI for the good.”

## Literature Review

Many have already studied the components of “AI for the good.” I review these attempts in four parts. First, I establish the precedent for practitioners to reflect on data science. Second, I summarize critiques of AI systems and language. Third, I review promising directions for the field. Fourth, I present attempts AI practitioners have made to improve elements of “AI for the good.”

First, following Agre (1997) and Iliadis and Russo (2016), I critically reflect on data science. I draw on science and technology studies and discursive analysis to bolster the integrity of scientific knowledge through “socially robust knowledge” (Nowotny, 2003). I speak to practitioners of AI as well as to those who study the use of such tools.

Second, existing works provide or analyze the meanings beyond the underlying functioning of AI systems. There are claims that these data-focused technologies might overcome theory (Anderson, 2008) or transform modern life (Mayer-Schönberger and Cukier, 2013). In examining “the algorithm as a thing and the algorithm as a word,” I choose words rather than the content of techniques as the site of critique (Beer, 2017, p. 9). Words are crucial because “by definition, a technological project is a fiction, since at the outset it does not exist, and there is no way it can exist yet because it is in the project phase” (Latour and Porter, 1996). “AI for social good” is one such project—if it already existed, why say so? Even AI alone, “evokes a mythical, objective omnipotence, but it is backed by real-world forces of money, power, and data” (Powles, 2018). Here Beer”s dichotomy between the algorithm as a word and as a physical manifestation becomes evident. Associating other words with AI—like intelligent, good, or society—creates notions of efficiency, neutrality, and progress, like how many technological metaphors (Stark and Hoffmann, 2019) “are myths that suffuse modern society” (Dalton and Thatcher, 2014) wielding power.

In rebutting common notions of neutrality from practitioners, Green focuses on the political nature of AI technologies. He thoroughly argues that data science should be seen of as political and, responding to the frequent practitioner argument that “We should not let the perfect be the enemy of the good,” states, “data science lacks any theories or discourse regarding what “perfect” and “good” actually entail” (Green, 2018, p. 19). The pro-technology argument takes “for granted that technology-centric incremental reform is an appropriate strategy for social progress” (Green, 2018, p. 19) without having to worry about how (or whether) this actually occurs. This belief that the introduction of a technology is sufficient to yield a positive end is often, like by Dalton and Thatcher (2014), called technological determinism.

Third, there are promising approaches to define “good” with regard to AI. Social work provides one application. Tambe and Rice propose a union between social workers and AI practitioners, because “AI can be used to improve society and fight social injustice” (Tambe and Rice, 2018, p. 3). Patton, a social worker academic, finds footing for such a union and identifies ways AI practitioners can engage well—largely by privileging those with whom they work (Patton, 2019). D'Ignazio adds to this by applying a social work code of ethics to data scientists, making explicit the principles to which data scientists seldom commit, like commitments to social justice and to the communities with whom they work (D'Ignazio, 2018).

Fourth, AI practitioners use terms like “AI for good” seemingly without regard to their notional or metaphorical value, but some engage with what might constitute “good.” Practitioners, like Niño et al. (2017), use “social good” as a *domain* from which to solve problems (“the field of social good”) (Niño et al., 2017, p. 896). These projects are designed for “serving the people who are in need globally, improving the society we live in and people's conditions within it” and make up application areas like health care, ecology, human rights, child welfare, etc. (Niño et al., 2017, p. 897). Niño et al. characterize key areas in projects for “social good” in a framework including data ownership, ethics, sustainability, assessment, stakeholder engagement, etc. Nevertheless, they do not mention what makes a project constitute “social good” except as existing in one of the application areas^16^, as described by Green”s and D'Ignazio”s critiques. Using “social good” as a domain risks allowing the constituent projects to be seen of as good even if they fail to meet principles espoused by others (like by having poor data management practices), use no principles at all, or, more importantly, meet a set of principles that actively violate the principles of social justice (but retain the term “good”). I will henceforth refer to this understanding of “social good” as the *domain definition*.

For example, Green questions the focus on crime prediction systems at USC's AI for Social Good initiative. He argues that the initiative bolsters racist and oppressive policing instead of working to address the structural problems which lead to police action (Green, 2018). Similarly, Palantir, a big data company that produces crime prediction systems for clients like the U.S. government, recently partnered with the United Nation's World Food Program (WFP) (World Food Program, 2019). One might argue that such an endeavor is “social good” given that WFP is a not-for-profit aimed at reducing poverty. Nonetheless, this partnership met a significant outcry from groups like the Responsible Data List (Easterday, 2019). Clearly, these groups interpret “social good” quite differently. Their disagreement indicates the insufficiency of the domain definition.

Other practitioners working in “AI for good” recognize limitations of their efforts. Researchers at IMB call for a shift to produce open AI platforms to mitigate one-off projects (Varshney and Mojsilovic, 2019). Maxmen questions the worth of the Big Data for Good project from a global telecommunications group in its use of call detail records to respond to disasters because governments might (mis)use the same data for surveillance (Maxmen, 2019). Along the same lines, but largely not using the term “AI for good,” recent work in fairness, accountability, and transparency (FAT^*^) has aimed to define best principles and practices for AI systems. Like Greene et al. (2019) and Lipton (2016) note that such technical efforts occur in too limited a manner; they present reforms to structures that might better be replaced. Selbst et al. expand on these critiques to note how FAT^*^ as a field misses the broader social context and might be better served focusing on process and collaborating deeply with domain experts (Selbst et al., 2019).

In an examination of the entire field of AI, as opposed to individual projects, Floridi et al. identify principles for the creation of a “good AI society” regarding under-use, mis-use, and over-use (Floridi et al., 2018). Improving on others, they use the term “AI for social good” just once and not in the context of a discipline, but rather to identify the application of their framework^17^. Notably, they focus on potential harms (like those possible from a general artificial intelligence) on an equal, if not greater, degree than current harms (like threats to individual privacy). This corresponds to their inclusion of under-use of AI as a risk. Given the current harms of AI, their “good AI society” may just be a “good bad society,” or, the best of the worst.

Prominent AI practitioners have acknowledged some of the inherent risks and ambiguities of AI technologies (Dietterich and Horvitz, 2015; Horvitz and Mulligan, 2015), but they do so in a way that appears to just pay lip service to, and thus avoid, fundamental critiques. To paraphrase, they argue that the risks of AI technologies are important, but that the risks can only be solved by further development of AI technologies. The utopic notion of economic liberalism employs the same sort of rhetoric: because the free-market ideal has never been achieved, one can always argue that its failures are due to insufficiently free markets (Polanyi, 2001). Likewise, data scientists, instead of addressing critiques, focus on how to realize the ideal of datafication in society (Rouvroy et al., 2013); they reinforce a technological determinism. In this way, the use of “AI for the good,” given the domain definition, appears to strategically avoid consideration that the risks of AI may be too great to consider any further development of the technologies.

Many arguments for and notions of AI technologies sit on lose ground. Critiques of these technologies highlight their limitations, often in the sense of technological determinism and the avoidance of structural problems. A greater focus on these political problems and an engagement with communities might reorient the field. With these in mind, I examine whether “AI for good” is appropriate to classify the field.

## The good and bad of “AI for good”

When working for “the good” we must ask *which* good and *for whom*. By committing to definitions of what constitutes “good” and “bad” with regard to AI technologies, I examine the appropriateness of labeling the field as “AI for the good.” I described in the literature review how the clearest criteria for “AI for good” is based on the domain with which an AI technology interacts (the domain definition). We are meant to accept that because a project works on health, with not-for-profit organizations, in the space of climate change, on poverty-reduction, etc., that it is “for social good.” In this section, my argument is as follows:

1. I provide an alternative definition of good according to the *capability approach* and social justice.
2. Following 1, there are projects that are good, but that are not labeled as such.
3. Following 1, AI technologies carry inherently bad externalities.
4. Following 3, in order to consider net goods, “AI for social good” must engage with and balance out these bads.

First, I offer a functional definition of “good” for an AI system using the capability approach and social justice. Green (2018, p. 4) cites (Collins, 2002) in defining a social justice project as “an organized, long-term effort to eliminate oppression and empower individuals and groups within a just society” and advocates for such projects in data science. Such a project can work in complement with the capability approach, a theoretical framework predicated on context-dependent individual freedom and well-being as defined by people's capabilities or real opportunities to act. This approach, particularly as evoked in the areas of information and communication technologies (Johnstone, 2007; Kleine, 2010), provides an operational lens for AI technologies. I use the capability approach with a particular focus on accountability and individual control over private information to highlight voices from historically marginalized communities Of course, one might disagree with my definition on many grounds—mine is neither radical (e.g., anti-capitalism) nor conservative (e.g., a defense of the status quo) enough and remains vague. My point is not so much to advance *this* definition as to advocate for discussion of *which* definition is most appropriate just as Green, Patton, and D'Ignazio do. Such a frame will then allow us to analyze claims of “social good.” Suffice to say, a “good” intervention should be empowering (particularly of basic human functioning), address structural conditions of oppression, and perform at least as well as interventions using similar amounts of resources.

For example, imagine a project designed in partnership with a community in a specific West-African country with little access to health care. The project uses a computer vision application on a smartphone to screen babies for birth defects. This project might be viewed of as “good” given that it specifically works with a marginalized community and increases their capability to access health care. Further, the community might not have achieved the same access to care with a similarly-resource-intensive effort to train more medical professionals.

Second, using the definition of good from 1, there are projects which do not use the label “AI for the good” that might be classified as such. For example, consider recent efforts in federated learning to decentralize and distribute the computations constituent in the training of a model (McMahan and Ramage, 2017). These efforts address some concerns about the privacy of user data: such data might not need to be collected in the same centralized manner. Furthermore, one can imagine a fully-specified federated learning project that meets the criteria of 1. Despite this, the concept of federated learning does not carry the moniker social good.

Third, inherently bad externalities arise with AI technologies. Recent work has shown that model training creates a significant carbon footprint (Strubell et al., 2019). In order to create an AI system, one must employ many engineers and scientists and set-up infrastructure, all of which are costly—perhaps more so than other interventions. Even more significantly, enormous invisible and unacknowledged labor goes into labeling data for training purposes, much of which occurs under potentially or explicitly exploitative conditions (Gray and Suri, 2019). Datafication names the creep to record more of life in a manner that can be processed by a computer (Mayer-Schönberger and Cukier, 2013). It undergirds the bloom in AI—models need data to combine with human labels—but brings unknown harms. Data collected for what 1 day appears good may be used later for what may not accord the same definition of good. For example, data to improve resource distribution to parolees were later used to create a model to gauge how likely offenders were to recommit crimes (Angwin and Larson, 2016). Datafication works at odds with user privacy as seen with consumer hacks, behavioral advertising, and government surveillance (Zuboff, 2019).

“*AI for good” distracts from the larger world in which AI exists*. Public visibility does not acknowledge the interdependent and exploitative nature of the technologies. Labeling them as “for the good” positions them as somehow intrinsically better than the social systems on which they depend. For example, tech companies implement systems they acquire from start-ups created from academic research. Most research papers come from graduate students whose long working hours are enabled by the labor of custodial staff and food service employees. In order to respond to questions on the appropriateness of a long short-term memory or a hidden Markov model one must not just understand their error rates, but also how to calculate derivatives, engage in basic math, and use language—skills learned through years of, for most, public schooling and from hundreds of teachers. AI models run on machines made thousands of miles away by people practitioners will never meet. These machines draw electricity produced by fossil fuel workers and which is distributed through a grid maintained by scores more. The startups themselves, or the tech companies that buy up startups to “scale” their systems, then farm out the process of data labeling to vast networks of invisible workers (Gray and Suri, 2019). To even have the capacity to build an AI system requires what Anderson describes of as “joint-production” (Anderson, 1999, p. 321). Those involved in AI systems are not just the visible actors of engineers, scientists, researchers, program managers, marketers, negotiators, lawyers, or end users. These terms too precisely assign agency, ability, and intentionality to what is best described as panning the sediment of streams of data.

The point is not to decry actors who lay claim to terms like “AI for the good” so much as to question how their actions reflect on their stated goals. Those who use such terms may even believe that they are saving the field of AI from “not good” domains, that their research areas are the more appropriate direction. Given the overheads and externalities of AI, it is not clear there is such a need at all to focus on “not good” domains. Even with criteria to label AI systems as “good,” the inherent interdependence raises questions about whether AI is inherently “bad” and whether any domain can redeem the system of production.

“*AI for the good” is strategically vague*. Left out by the use of “AI for the good” is the intensely political nature of any one of the areas associated with the term (as in domain definition). Recall the USC AI for Social Good project on policing which Green named as oppressive. Indeed, according to the definition of good from 1, the USC project would be bad—it does privilege community voices and reinforces forms of oppressive policing (which restrict peoples” capabilities).

Furthermore, non-profit organizations, which at least some AI practitioners associate with their use of “social good,”^18^ might not even desire such technology. For example, for these non-profits, technical contributions might be better spent on upgrading old systems (like from Windows XP) rather than spending resources to get data in the “right” format for building AI systems.

Fourth, this all suggests that to be considered “good,” projects must commit to a definition of social good and then show that, even after considering negative externalities, on the balance they still achieve good. On the whole, then, projects might better consider the degree to which they are “not bad.”

## Critiques

In this section, I consider four critiques of my argument.

First, detractors might chafe at a focus on the words of AI. They might argue that focusing on words ignores the substance of technologies which would actually bring about “good.” Of course the substance of the technologies is important, but in this paper I focus on the use of language, which, as I make the case for in the introduction, is also important.

Second, one might posit that even if “AI for the good” is vague, the use of such terms does no harm. While the claim of vagueness has been used to decry the difficulty of regulating AI technologies (Scherer, 2015), we use vague terms like energy or manufacturing and are able to operationalize them (Danaher, 2018). In this sense, the absence of a definition would be permissible so long as we “know it when we see it.” This is not the case with claims of “social good.” Such a response is strategically vague; it elides the externalities inherent to AI technologies and uses the weak criteria of the domain definition. Harm comes in allowing ourselves to feel good while perpetuating oppressive systems and when misallocating resources.

Third, a reader might say that “social good” is just marketing speak—not what practitioners say. That may be so, but the term appears from research to implementation: in governments, in funding agencies, in research papers, at conferences, in companies, and in public discourse. Even if the majority of the use of “AI for good” occurs externally to AI practitioners, it is through these routes that the notions of AI manifest. That is, practitioners must care about how their work is used and not just what it is.

Still, one might argue that, fourth, despite its flaws, “social good” is a relevant distinction. Even in the absence of a more robust criteria, there is a difference between machine learning researchers choosing to work on credit card companies being defrauded vs. those working on disease modeling. I suggest that there is a better approach than to ignore the ambiguity, the insufficient criteria, and the externalities of AI. Instead of banishing “AI for good,” we might rather rename the field.

## Suggestions

In this critique of the use of language, I also offer a suggestion. Namely, we should stop labeling projects as “for social good” and instead use the term “for not bad.” The latter more accurately evokes the need to avoid the inherent bad traits of AI technologies without falling into the traps involved with vague claims to “social good.”

Practitioners who would still like to use terms like “AI for the good” should read literature that studies the criteria for evaluation of social change projects and then apply those criteria. This includes work in the health sciences, social sciences, development studies, economics, and more. In the scope of technological changes for implementing theories of a just society, the literature in Information and Communication Technologies for Development provides some examples. Conferences in this space include the ACM Conference on Computing and Sustainable Societies (COMPASS) and the Workshop on Computing Within Limits^19^. The journal *Information Technology and International Development* focuses on the background theory of such work^20^.

With such a background, practitioners may be better prepared to define and measure criteria of “good” to expand on my attempt above. More work to quantify the externalities of AI projects [building on examples like Maxmen (2019) and Strubell et al. (2019)] will then fill out such criteria. This might include comparable metrics on cost, energy usage, and potential for future misuse of data.

Sustained interaction with those in communities that are to be “innovated” will further concretize what constitutes “good.” Tambe and Rice (2018) and Patton (2019) demonstrate how this can be done with social work. Action research, like as related to human computer interaction by Hayes (2011), provides another lens for community interaction in terms of accountability and shared credit for results. Many “social good” initiatives already discuss a focus on partnerships^21^—these should be expanded and made sure to recognize, if not attempt to address, the underlying structural issues.

“AI for not bad” avoids some of the problems of “AI for good.” It more honestly describes the current vagueness and centers the externalities. Practitioners unwilling or unable to commit to explicit notions of good should consider adopting it.

## Conclusion

“AI for the good” is vague, lacks sufficient criteria, omits the externalities of AI, and elides the structural interdependence of AI projects. “AI for the good” may really be AI for flashy slide decks, AI for difficult-to-maintain and highly interdependent computational systems, AI for new statistical methods, or (at best) AI for public health analyses that may end up saving lives. In this paper, I raise concerns about the presentation of the “AI frontier” as beneficent. Following Green, I ask that the field “AI for the good” recognize that, as it is now, it really constitutes “AI for not bad.” Practitioners would more honestly embrace this label or else do the work necessary to legitimately claim good.

In this work, I advocate for a more honest discipline. I ask those out there who interact with AI at any level—the new student wondering where to put her time, the executive of a company—to consider what their use of language ignores.

“AI for social good” speaks to the desire of many of practitioners to share what opportunities they have. It sounds nice. It imagines a world of lucrative careers optimized to better humanity. The world is not so simple. Perhaps it is enough that society, as bolstered by science, has tended toward longer lives, more food, and less violence (Pinker, 2019; Rosling et al., 2019), but extrapolation will not alone resolve problems. AI practitioners, like myself, are part of the prospecting of science from which we hope for gold, but in which will we likely find just sand—and perhaps leave in our tailings environmental damage and labor displacement. Lest that be so, we must be honest about what we are doing and what we might do better.

## Author Contributions

JM contributed everything to this paper and is accountable for the content of the work.

### Conflict of Interest Statement

The author declares that the research was conducted in the absence of any commercial or financial relationships that could be construed as a potential conflict of interest. The handling editor and reviewer HL declared their involvement as co-editors in the Research Topic, and confirm the absence of any other collaboration.

## Footnotes

^1^https://research.cornell.edu/research/human-compatible-ai; https://humancompatible.ai

^2^https://hai.stanford.edu/

^3^https://caire.ust.hk/about/about-caire

^4^https://dssg.uchicago.edu/; https://escience.washington.edu/dssg/

^5^https://cyber.harvard.edu/story/2018-12/embracing-ai-social-good; https://www.cais.usc.edu/about/mission-statement/

^6^https://aiforsocialgood.github.io/2018; https://aiforsocialgood.github.io/icml2019/; https://aiforsocialgood.github.io/iclr2019/

^7^https://www.bloomberg.com/company/d4gx/; https://www.kdd.org/kdd2014/bloombergpress.html

^8^http://iaenmissionsociale.com/

^9^https://www.re-work.co/events/ai-for-good-summit-san-francisco-2019

^10^https://psa2018.philsci.org/en/74-program/program-schedule/program/124/psa2018-public-forum-for-the-public-good-values-and-accountability-in-ai-and-data-science

^11^https://ai.google/social-good; https://fbaiforindia.splashthat.com; https://www.ibm.com/watson/advantage-reports/ai-social-good.html; https://www.intel.ai/ai4socialgood/

^12^https://www.microsoft.com/en-us/ai/ai-for-good

^13^https://allenai.org/; http://wadhwaniai.org/; https://mila.quebec/en/ai-society/

^14^https://www.niti.gov.in/writereaddata/files/document_publication/NationalStrategy-for-AI-Discussion-Paper.pdf; https://www.whitehouse.gov/ai/; http://www.baai.ac.cn/blog/beijing-ai-principles

^15^Schank (1987) and Bringsjord and Schimanski (2003) allow that AI exists given a narrow functional definition and explore the complications of intelligence.

^16^Their framework does address a “lack of standardized good practices to leverage the power of data” (Niño et al., 2017, p. 897).

^17^“This should involve a clear mission to advance AI for social good, to serve as a unique counterbalance to AI trends with less focus on social opportunities” (Floridi et al., 2018, p. 704).

^18^https://www.research.ibm.com/science-for-social-good/

^19^https://acmcompass.org/; http://computingwithinlimits.org

^20^https://itidjournal.org/index.php/itid

^21^For example, WadhwaniAI partners with India's Central Tuberculosis Division https://wadhwaniai.org
